# Circulating activated lymphocyte subsets as potential blood biomarkers of cancer progression

**DOI:** 10.1002/cam4.3150

**Published:** 2020-05-27

**Authors:** Ying‐Yi Wang, Na Zhou, Hong‐sheng Liu, Xiao‐Lei Gong, Rui Zhu, Xiao‐Yuan Li, Zhao Sun, Xu‐Hong Zong, Ning‐Ning Li, Chang‐Ting Meng, Chun‐Mei Bai, Tai‐Sheng Li

**Affiliations:** ^1^ Department of Medical Oncology Peking Union Medical College Hospital Chinese Academy of Medical Sciences and Peking Union Medical College Beijing China; ^2^ Department of Thoracic surgery Peking Union Medical College Hospital Chinese Academy of Medical Sciences and Peking Union Medical College Beijing China; ^3^ Department of Medical Record Peking Union Medical College Hospital Chinese Academy of Medical Sciences and Peking Union Medical College Beijing China; ^4^ Department of Medical Oncology Wangshi Town Hospital Haicheng China; ^5^ Institute for Systems Biology Seattle WA USA

**Keywords:** cancer development, cancer progression, clinicopathologic characteristics, lymphocyte subsets, marker, peripheral blood

## Abstract

The objective of this study was to predict the value of lymphocyte subsets in cancer progression. Peripheral blood was obtained from 327 untreated patients with cancer and 158 healthy volunteers. Levels of lymphocyte subsets were determined by flow cytometry. There were decreased levels of natural killer (NK) cells, CD8^+^ T cells, and naïve CD4^+^/CD4^+^ T cells in untreated patients with cancer compared to those in healthy controls. Inversely, there were elevated levels of the following T‐cell percentages in cancer patients compared to those in healthy controls: memory CD4^+^/CD4^+^, CD8^+^ T cells, HLA‐DR/CD8^+^, CD8^+^ CD38^+^/CD8^+^, and CD4^+^/CD8^+^. In addition, there are a decreasing trend in terms of CD4^+^ T‐cell counts and an increase CD8^+^ HLA‐DR/CD8^+^ T‐cell and CD8^+^ CD38^+^/CD8^+^ T‐cell percentages in the advanced stage. An increasing trend with advanced tumor stage and the percentages of CD8^+^ HLA‐DR/CD8^+^ T cells and CD8^+^ CD38^+^/CD8^+^ T cells was shown in this study. There are a negative correlation for CD4^+^ T‐cell counts and positive correlation for percentages of CD8^+^ HLA‐DR/CD8^+^ T cell and CD8^+^ CD38^+^/CD8^+^ T cells with the lymph node metastasis. In the presence of distant metastatic spread, we observed higher NK‐cell counts, CD8^+^ HLA‐DR/CD8^+^ T‐cell percentages, CD8^+^ CD38^+^/CD8^+^ T‐cell percentages, as well as lower CD4^+^ T‐cell counts than those in the absence of distant metastases spread. Abnormal levels of NK cell, CD8^+^ T cells, memory CD4^+^/CD4^+^, naïve CD4^+^/ CD4^+^, CD8^+^ HLA‐DR/CD8^+^, CD8^+^ CD38^+^/CD8^+^, and CD4^+^/CD8^+^ can be a potential blood biomarkers of cancer development. CD4+ T‐cell counts and percentages of CD8^+^ HLA‐DR/ CD8^+^ and CD8^+^ CD38^+^/ CD8^+^ can predict the cancer progression.

## Graphical abstract

Abnormal levels of NK cell, CD8^+^ T cells, memory CD4^+^/CD4^+^, naïve CD4^+^/CD4^+^, CD8^+^ HLA‐DR/CD8^+^, CD8^+^CD38^+^/CD8^+^, and CD4^+^/CD8^+^ can be a potential blood biomarkers of cancer disease progression. Lymphocyte subsets levels were related to gender, age, stage, tumor stage, lymph node metastasis, distant metastasis, and differentiation.

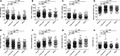

## 1 INTRODUCTION

The global burden of cancer continues to significantly increase due to the growth and aging of the worldwide population, as well as the increasing adoption of cancer‐causing lifestyle factors such as smoking, poor diet, and sedentary behavior.^1^

The tumor microenvironment includes immune cells (macrophages, mast cells, myeloid‐derived suppressor cells, neutrophils, dendritic cells, T cells, and B cells), tumor cells, and the surrounding stroma.^2^ Lymphocyte subsets are crucial in regulating immunity and specific killing of tumor cells and are closely associated with the development of solid tumors due to their diverse roles in immune responses.^3^ As a subset of lymphocytes of the innate immune system, NK cells monitor cell surfaces of autologous cells in the absence of antibodies or major histocompatibility complex (MHC) class I molecules and participate in adaptive immune responses similar to cytotoxic T cells.^4^ CD4^+^ T‐cell subsets are involved in the development and maintenance of the adaptive immune system. There are various subtypes of CD4^+^ T cells, as reflected by their diverse functions.^5^ CD4^+^ T cells can secrete interferon (IFN)‐γ, activate other lymphocyte subsets by releasing T‐cell cytokines, and suppress tumor development by directly killing tumor cells expressing adequate levels of MHC class II molecules.^6^ As cytotoxic cells, CD8^+^ T‐cell subsets are able to recognize tumor cells by presentation of tumor‐associated antigens in complex with MHC class I molecules and produce IFN‐γ for targeting and killing of cancer cells.^7^

The levels and roles of classical lymphocyte subsets including CD4^+^ T cells, CD8^+^ T cells, and CD45RA T cells have been observed in patients with breast cancer, advanced oral cancer, ovarian cancer, myeloma, head, and neck cancer, and liver cancer.^8^, ^9^ However, there is no consensus on the different levels of lymphocyte subsets in cancer progression. Recent studies have indicated that lymphocyte subsets are related to gender, age, and disease stage in cancer patients.^10^, ^11^ Levels of lymphocyte subsets differ significantly by gender. The decline in the normal function of the immune system associated with aging is called immunosenescence. Immunosenescence can increase the risk of infection, cancer, and autoimmune diseases.^12^ Although previous reports have confirmed a relationship between lymphocyte subsets and clinicopathological parameters, the details of these associations remain to be established.

The aim of our study was to evaluate potential blood biomarkers of T‐cell subsets in cancer disease progression and the clinical response to immune therapy, and the relationships between these levels and clinicopathological parameters in untreated cancer patients. These potential biomarkers might contribute to immune dysfunction in untreated cancer patients.

## 2 MATERIALS AND METHODS

### 2.1 Patients and clinical data

Participants (n = 485) were recruited at the Peking Union Medical College Hospital between February 2007 and May 2019. A total of 327 cancer patients who did not receive any treatment (174 men and 153 women) aged 18‐84 years (median age: 58.98 years) were enrolled. Patients with viral infection were excluded because the infection might have affected lymphocyte subsets levels. The patient group included 199 lung cancer patients, 68 colon cancer patients, 20 gastric cancer patients, 9 esophageal cancer patients, 6 breast cancer patients, 5 thymic carcinoma patients, 5 pancreatic cancer patients, 4 head and neck cancer patients, and 11 patients with other cancers. Patient clinical data are summarized in Table 1. A total of 158 age‐ and gender‐matched healthy volunteers (96 men and 62 women) were selected with ages ranging from 19 to 80 years (median age: 58.77 years). Age was divided into three groups according to the World Health Organization (young: 0‐44 years; middle age people: 45‐59 years; and elderly people: over 59 years). Informed consent was obtained from all participants. This study was approved by the Ethical Committee of Peking Union Medical College Hospital.^13^

**TABLE 1:** Characteristics of study patients

	Lung cancer (n = 199)	Colon cancer (n = 68)	Others (n = 60)	Total (n = 358)
Gender				
Male	96	39	39	174
Female	103	29	21	153
Age				
Yong	14	5	12	31
Middle	76	22	21	119
Elder	109	41	27	177
ECOG PS				
0	186	50	34	270
1	5	9	13	27
2	0	1	2	3
Unknown	8	8	11	27
Stage				
I	133	7	5	145
II	10	4	5	19
III	19	12	7	38
IV	33	41	32	106
Unknown	4	4	11	19
Tumor stage				
T1	129	1	6	136
T2	38	9	4	51
T3	9	16	4	29
T4	10	10	11	31
Unknown	13	32	35	80
Lymph nodes				
N0	140	13	7	160
N1	7	12	4	23
N2	25	10	9	44
N3	11	0	5	16
Unknown	16	33	35	84
Distant metastases				
M0	162	23	17	202
M1	33	41	33	107
Unknown	4	4	10	18
Differentiation				
Poorly	10	8	18	36
Middle	14	38	8	60
High	118	13	4	135
Unknown	57	9	30	96

Abbreviation: ECOG PS, Eastern Cooperative Oncology Group (ECOG) performance status.

### 2.2 Lymphocyte immunophenotyping

Lymphocyte immunophenotyping was performed using three‐color flow cytometry (Epics XL flow cytometry; Beckman Coulter, USA) as previously described.^14^ The percentages and counts of the following lymphocyte subsets were measured, including CD3‐CD16^+^ CD56^+^ NK cells, CD4^+^ T cells, CD8^+^ T cells, memory CD4^+^ T cells, CD4^+^ CD45RA+CD62^+^ naïve T cells, CD8^+^ HLA‐DR T cells, CD8^+^ CD38^+^ T cells, and the CD4/CD8 ratio. The memory CD4^+^ T cells were counted by difference between CD4 T cells and naïve CD4^+^ T cells. Freshly collected thylenediaminetetraacetic acid and (EDTA)‐anticoagulated whole blood were incubated and tested with a panel of monoclonal antibodies directed against fluorescein isothiocyanate/phycoerythrin/peridinin chlorophyll protein combinations of CD3 (clone:SK7)/CD8 (clone:SK1)/CD4 (clone:SK3), CD3/CD16(clone:B73.1)/CD56 (clone: NCAM16.2), HLA‐DR(clone:SK7)/ CD38(clone:SK7)/ CD8, CD62L(clone:SK11)/ CD45RA (clone:L48)/CD4, and isotype controls (Immunotech, France). Cell counts of lymphocyte subsets were calculated using a dual‐platform method with white blood cell counts and lymphocyte differentials obtained from routine blood tests of the same specimen. The strategies for different cell subsets are shown in Figure S1.

### 2.3 Statistical analysis

Statistical analysis was performed using SPSS 21.0 software (IBM Corporation). The Shapiro‐Wilk test was used to assess the normality of distributions. Reference ranges were calculated using the mean ± standard deviation (SD). *T* tests and one‐way analysis of variance were used for parametric data. Mann‐Whitney and Kruskal‐Wallis tests were used for nonparametric data. Correlational analyses were performed using the Spearman's rank correlation test. Probability values were derived from two‐sided tests and values of *P* < .05 were considered statistically significant. Figures were prepared using GraphPad Prism 7.0 software (San Diego, USA).

## 3 RESULTS

### 3.1 Levels of NK cells, and the following T cells differed between all cancer patients and healthy volunteers: CD8^+^, memory CD4^+^/CD4^+^, naïve CD4^+^/CD4^+^, CD8^+ ^HLA‐DR/CD8^+^, CD8^+ ^CD38^+^/CD8^+^, and CD4^+^/CD8^+^

To explore the predictive value of lymphocyte subset levels in untreated cancer, a total of 485 Chinese adults were enrolled in this study. Levels of lymphocyte subsets in cancer patients were compared with healthy controls. The results are shown in Table 2. Several reports have confirmed that levels of observed lymphocyte subsets were significantly associated with gender and age in healthy individuals and cancer patients; thus, we carefully avoided age‐ and gender‐related biases. NK‐cell counts (*P* < .001), CD8^+^ T‐cell counts (*P* < .001), and naïve CD4^+^/ CD4^+^ T‐cell percentages (*P* < .001) were lower in cancer patients than in healthy controls. In contrast, a higher percentage of memory CD4^+^/ CD4^+^ T cells (*P* < .001), CD8^+^ HLA‐DR/ CD8^+^ T cells (*P* < .001), CD8^+^ CD38^+^/ CD8^+^ T cells (*P* < .001), and the CD4^+^/ CD8^+^ ratio (*P* < .001) were observed in cancer patients compared to healthy controls. CD4^+^ T‐cell levels did not differ significantly between patients and controls (*P* > .05).

**TABLE 2:** Comparison of lymphocyte subsets levels in cancer patients and healthy controls

Lymphocyte subsets	Healthy controls (N = 327)	Healthy controls (N = 158)	*P* value
NK cell (cells/uL)	396.194 ± 244.44	275.68 ± 209.28	**<.001**
CD4^+^ T cell (cells/uL)	699.73 ± 253.23	736.11 ± 289.36	.198
CD8^+^ T cell (cells/uL)	536.25 ± 272.92	441.12 ± 212.37	**<.001**
Memory CD4^+^/ CD4^+^ (%)	67.16 ± 13.57	74.05 ± 12.71	**<.001**
Naïve CD4^+^/ CD4^+^ (%)	32.85 ± 13.57	24.07 ± 12.77	**<.001**
CD8^+^ HLA‐DR/CD8^+^ (%)	28.72 ± 11.24	41.30 ± 15.30	**<.001**
CD8^+^ CD38^+^/CD8+ (%)	23.02 ± 15.65	35.62 ± 15.59	**<.001**
CD4^+^/CD8^+^ (%)	1.56 ± 0.85	2.02 ± 2.01	**<.001**

Note

Data were expressed as means ± SD.

### 3.2 Levels of NK cells, and the following T cells differed between patients with various cancer types and healthy volunteers: CD8^+^, memory CD4^+^/ CD4^+^, naïve CD4^+^/ CD4^+^, CD8^+ ^HLA‐DR/CD8^+^, CD8^+ ^CD38^+^/CD8, and CD4^+^/CD8^+^

**FIGURE 1:**
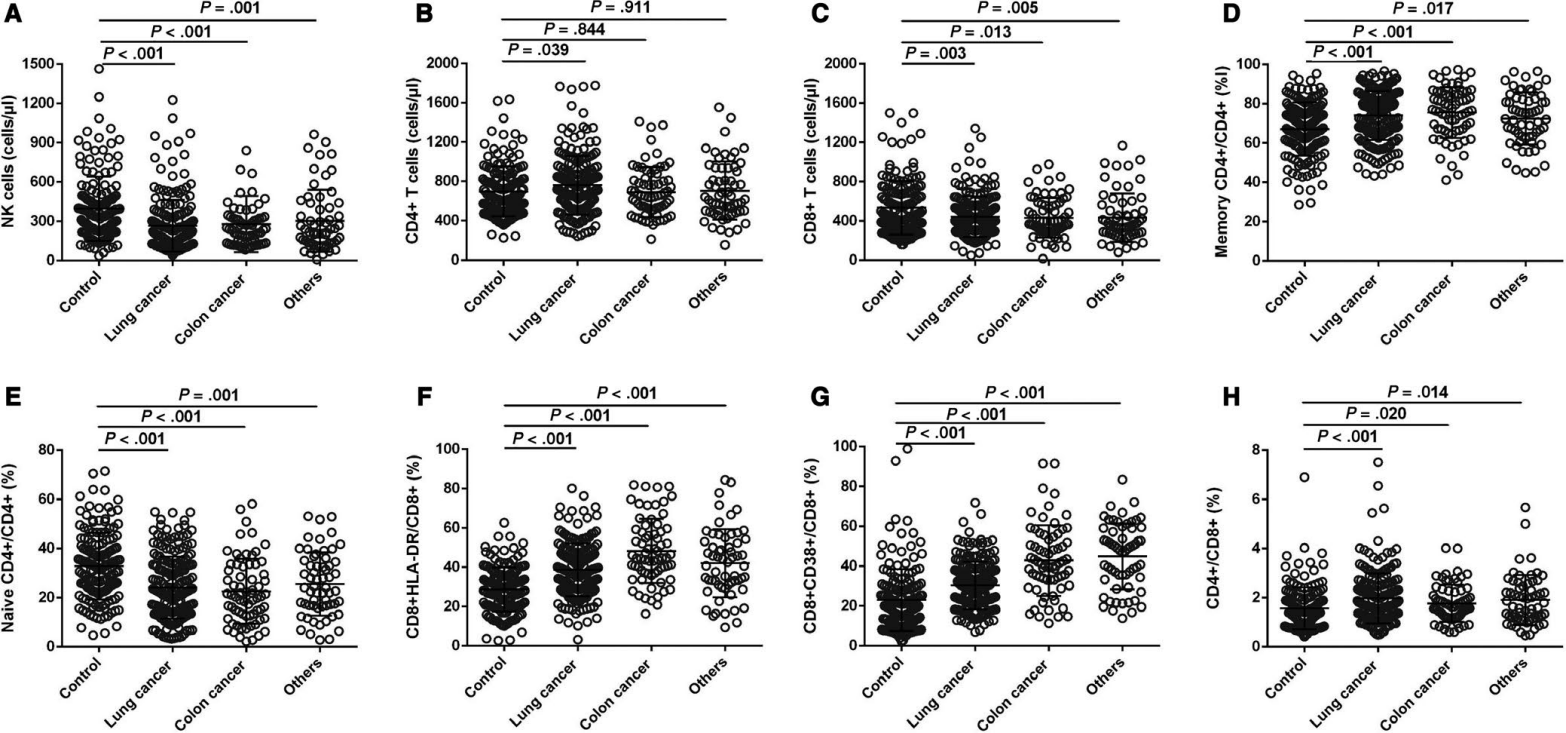
Predictive values of lymphocyte subsets levels in various cancers. A, Distribution of NK‐cell counts in four groups. B, Distribution of CD4^+^ T‐cell counts in four groups. C, Distribution of CD8^+^ T‐cell counts in four groups. D, Distribution of memory CD4^+^/CD4^+^ percentage in four groups. E, Distribution of naïve CD4^+^/CD4^+^ percentage in four groups. F, Distribution of CD8^+^ HLA‐DR/CD8^+^ percentage in four groups. G, Distribution of CD8^+^ CD38^+^/CD8^+^ percentage in four groups. H, Distribution of CD4^+^/CD8^+^ ratio in four groups

To further explore the predictive value of lymphocyte subset levels in various cancer types, patients were divided into three groups based on cancer type and size. The results for a single cancer were similar to those for all cancers (Figure 1). We observed significantly decreased NK‐cell counts (Figure 1A), CD8^+^ T‐cell counts (Figure 1C), and naïve CD4^+^/CD4^+^ T‐cell percentages (Figure 1E) in all cancer types compared to healthy controls. There was also an increased percentage of memory CD4^+^/CD4^+^ T cells (Figure 1D), CD8^+^ HLA‐DR/CD8^+^ T cells (Figure 1F), CD8^+^ CD38^+^/CD8^+^ T cells (Figure 1G), and CD4^+^/CD8^+^ T cells (Figure 1H). In addition, we only observed high CD4^+^ T‐cell counts in patients with lung cancer compared to controls (Figure 1B).

### 3.3 Correlations of levels of NK cells, CD4^+^ T cells, CD8^+ ^HLA‐DR/ CD8^+ ^T cells, and CD8^+ ^CD38^+^/ CD8^+^ T cells with cancer stage, tumor stage, lymph node metastases, and distant metastases

**FIGURE 2:**
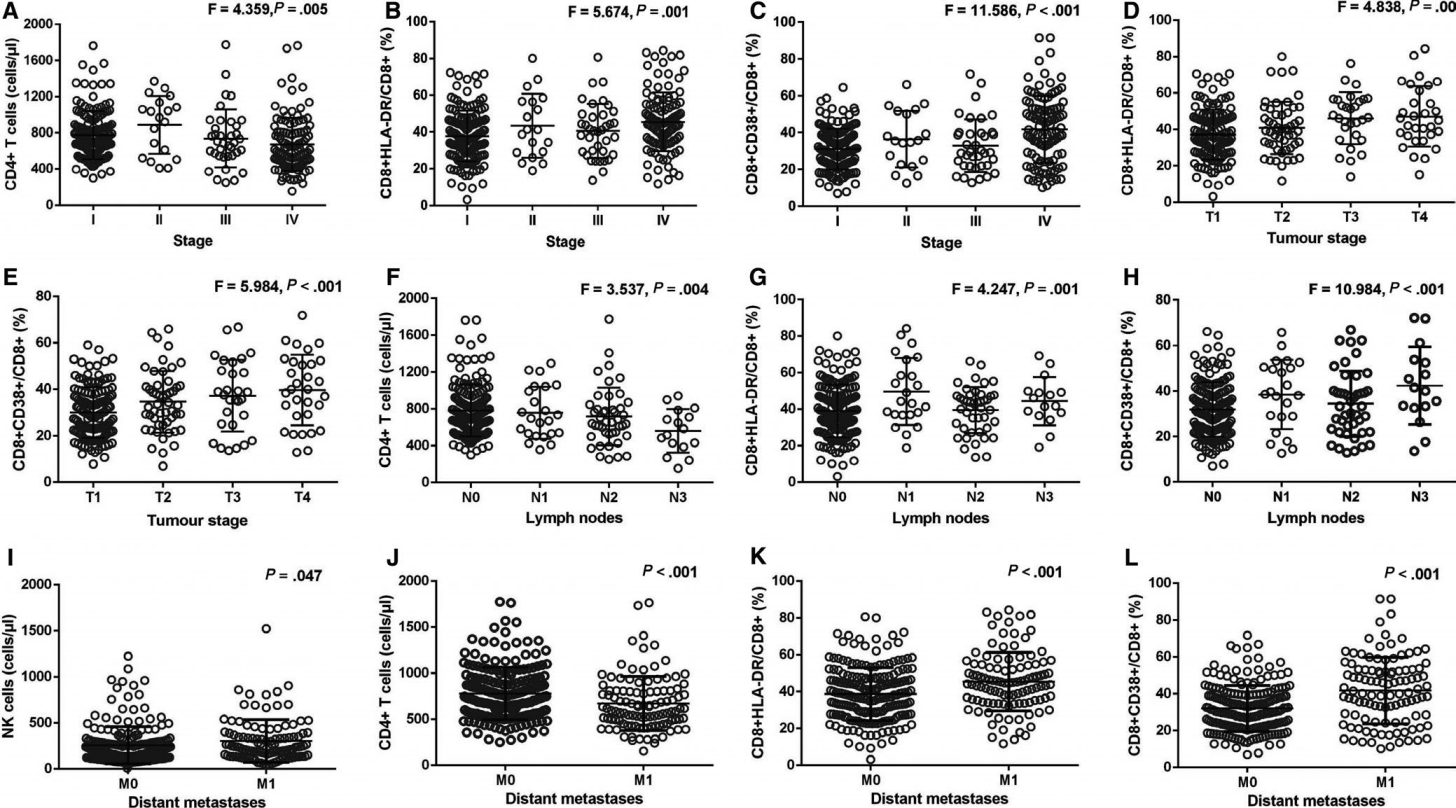
Relationships between levels of lymphocyte subsets and stage. A, Distribution of CD4^+^ T‐cell counts in patients at different stages; B, Distribution of CD8^+^ HLA‐DR/CD8^+^ percentage in patients at different stages; C, Distribution of CD8^+^ CD38^+^/CD8^+^ percentage in patients at different stages; D, Distribution of CD8^+^ HLA‐DR/ CD8^+^ percentage in patients at different tumor stages; E, Distribution of CD8^+^ CD38^+^/CD8^+^ percentage in patients at different stages; F, Distribution of CD4^+^ T‐cell counts in patients with or without lymph node metastases; G, Distribution of CD8^+^ HLA‐DR/CD8^+^ percentage in patients with or without lymph node metastases; H, Distribution of CD8^+^ CD38^+^/CD8^+^ percentage in patients with or without lymph node metastases; I, Distribution of NK‐cell counts in patients at distant metastases; J, Distribution of CD4^+^ T‐cell counts in patients at distant metastases; K, Distribution of CD8^+^ HLA‐DR/CD8^+^ percentage in patients at distant metastases; L, Distribution of CD8^+^ CD38^+^/CD8^+^ percentage in patients at distant metastases

To further explore predictive value of lymphocyte subset levels in cancer progression, we assessed associations between lymphocyte subsets and stage. The results are shown in Figure 2. A decreasing trend with advancing cancer stage in terms of CD4^+^ T‐cell counts (F = 4.359, *P* = .005, Figure 2A) was shown in our study. However, there was an increase in the advanced stage of CD8^+^ HLA‐DR/CD8^+^ T‐cell percentages (F = 5.674, *P* = .001, Figure 2B) and CD8^+^ CD38^+^/CD8^+^ T‐cell percentages (F = 11.586, *P* < .001, Figure 2C). An increasing trend with advanced tumor stage and the percentages of CD8^+^ HLA‐DR/CD8^+^ T cells (F = 4.838, *P* = .001, Figure 2D) and CD8^+^ CD38^+^/CD8^+^ T cells (F = 5.984, *P* < .001, Figure 2E) was shown in this study. There was a decrease in the lymph node metastasis‐related trend of CD4^+^ T‐cell counts (F = 3.537, *P* = .004, Figure 2F), but an increased lymph node metastasis‐related trend of CD8^+^ HLA‐DR/ CD8^+^ T‐cell percentages (F = 4.247, *P* = .001, Figure 2G) and CD8^+^ CD38^+^/CD8^+^ T cells (F = 10.984, *P* < .001, Figure 2H). In the presence of distant metastatic spread, we observed higher NK‐cell counts (*P* = .047, Figure 2I), CD8^+^ HLA‐DR/ CD8^+^ T‐cell percentages (*P* < .001, Figure 2J), CD8^+^ CD38^+^/ CD8^+^ T‐cell percentages (*P* < .001, Figure 2K), as well as lower CD4^+^ T‐cell counts (*P* < .001, Figure 2L) than those in the absence of distant metastases spread. There are no clear relationships among levels of memory CD4^+^/CD4^+^ T cells, naïve CD4^+^/CD4^+^ T cells, CD8^+^ T cells, or the CD4^+^/CD8^+^ ratio and age, stage, tumor stage, and lymph node metastasis differentiation.

### 3.4 Correlations of levels of NK cells, CD4^+ ^T cells, memory CD4^+^/ CD4^+ ^T cells, naïve CD4^+^/ CD4^+ ^T cells, CD8^+ ^HLA‐DR/ CD8^+ ^T cells, and CD8^+ ^CD38^+^/ CD8^+^ T cells with gender, age, and differentiation

To identify factors that might affect lymphocyte subsets, we assessed associations between lymphocyte subsets and clinicopathological characteristics. The results are shown in Figure S2. In female patients, we observed lower NK‐cell counts (*P* = .001, Figure S2A), memory CD4^+^/CD4^+^ T‐cell percentages (*P* = .025, Figure S2B), and higher naïve CD4^+^/CD4^+^ T cells (*P* = .022, Figure S2C) than those in male patients. A trend of increased percentages of memory CD4^+^/CD4^+^ T cells (*r* = .143, *P* = .009, Figure S2D) and CD8^+^ HLA‐DR/CD8^+^ T cells (*r* = .194, *P* < .001, Figure S2E) was noted for patients with increased age. An decreased trend with aging in CD8^+^ CD38^+^/CD8^+^ percentage (*r* = −.210, *P* < .001, Figure 2F) was shown in our study. A trend of increased CD4^+^ T‐cells counts (F = 3.191, *P* = .043, Figure S2G) and decreased CD8^+^ CD38^+^/CD8^+^ T‐cell percentages (F = 8.188, *P* < .001, Figure S2H) was observed with advancing differentiation. Interestedly, there were significant differences in CD8^+^ HLA‐DR/CD8^+^ T‐cell percentages (F = 3.254, *P* = .040, Figure S2I) for patients at various levels of differentiation; however, the highest value was observed in the middle stage of differentiation. In addition, there are no significant difference for the age and gender in different groups including the stage, tumor stage, lymph node metastasis, and distant metastases spread (*P* > .05). There were no clear relationships between CD8^+^ T‐cells counts or the CD4^+^/CD8^+^ ratio and gender, age, and differentiation. We did not analyze the relationship between ECOG and lymphocyte subsets because the samples size for ECOG was small.

## 4 DISCUSSION

In this study, we demonstrated that decreased levels of NK cells, CD8^+^ T cells, naïve CD4^+^/CD4^+^ T cells, and elevated percentages of the following T cells were associated with cancer incidence: memory CD4^+^/CD4^+^, CD8^+^ HLA‐DR+/CD8^+^, CD8^+^ CD38^+^/CD8^+^, and CD4^+^/CD8^+^. CD4^+^ T‐cell counts, and percentages of CD8^+^ HLA‐DR/CD8+, CD8^+^ CD38^+^/CD8^+^ are associated with the cancer progression. To our knowledge, this is the first study to investigate the predictive value of lymphocyte subsets in untreated patients with cancer.

### 4.1 NK cells in cancer

NK cells function in tumor immunosurveillance to monitor cancer cells and control local tumor growth by rapidly killing those cells without deliberate immunization or activation.^15^ Several studies have reported that patients had higher levels of NK cells than healthy controls, including patients with breast cancer and pancreatic ductal adenocarcinoma, which may be mixed with a considerable proportion of anergic NK cells.^16^, ^17^ More studies have demonstrated the relationship between low NK‐cell counts and increased cancer risk.^18^, ^19^, ^20^ Similarly, lower counts of NK cells were also observed in cancer patients compared with healthy controls in our study, which suggests that the function of NK cells was weaker for killing tumor cells, leading to oncogenesis.

### 4.2 CD8^+^ **T‐cell subsets in cancer**

CD8^+^ T cells, as cytotoxic cells, recognize antigens present in the context of MHC class I molecules and secrete cytolytic granules and chemokines to destroy cancer cells.^21^ In this study, decreased CD8^+^ T‐cell counts were observed in cancer patients compared with controls, which might imply that a low number of CD8^+^ T cells in peripheral blood reduces the ability to fight against tumors. The same result was demonstrated in another study.^22^, ^23^ However, conflicting results have also been reported in other studies.^24^ In the tumor microenvironment, CD8^+^ T cells undergo a period of massive expansion, activation, differentiation into effector cells, and apoptosis, which may lead to these disparate results.

As markers of CD8^+^ T‐cell activation, expression of HLA‐DR and CD38 plays a crucial predictive role in CD8^+^ T‐cell activation and CD4^+^ T‐cell depletion.^25^ Loss of HLA‐DR expression can induce tumor escape from immunosurveillance.^26^, ^27^ CD38 plays dual roles as a receptor and ectoenzyme. This molecule regulates the activation and proliferation of T lymphocytes. A high percentage of CD38^+^/ CD8^+^ T cells can predict disease progression and immunosuppressive status.^28^ In agreement with previous reports, we discovered that there were high CD8^+^ HLA‐DR+/CD8^+^ and CD8^+^ CD38^+^/CD8^+^ T‐cell percentages in patients with all cancer types compared with controls, suggesting that the immune system was activated during carcinogenesis.^29^

In this study, we discovered that CD8^+^ HLA‐DR+/ CD8^+^ and CD8^+^ CD38^+^/CD8^+^ percentage was positively correlated with cancer stage, tumor stage, lymph node metastasis, and distant metastasis. These results may suggest that antitumor response was activated with advancing cancer.

### 4.3 CD4^+^ **T‐cell subsets in cancer**

CD4^+^ T‐cell subsets play a dual role in immune responses. CD4^+^ T‐cell subsets can differentiate into various subsets with specific functions and properties, from the cytotoxic cell response stimulating Th1 and CD4^+^ cytotoxic T cells, to immune suppressing regulatory T cells.^30^ These cells can enhance and sustain cytotoxic CD8^+^ T‐cell responses during both the primary and secondary phases of the immune response. Simultaneously, they also have the ability to induce cytotoxic cellular immune responses.

CD4^+^ T cells, as cytotoxic cells, contribute to additional immune surveillance features.^31^ CD4^+^ T cells can broaden antitumor responses of CD8^+^ T cells and improve clinical responses.^32^ Luo demonstrated that patients with lung cancer have greater CD4^+^ cells than those in the healthy control group.^33^ Liu found similar results in hepatocellular carcinoma.^22^ However, conflicting results have been reported in other studies. Sheng demonstrated that there are no statistical differences in the peripheral blood of lung cancer patients compared with healthy controls.^34^ Huang et al reported similar results in multiple myeloma patients.^35^ These results are consistent with that of our study. CD4^+^ T cells amplified the response of cytotoxic T cells. Insufficient numbers of CD4^+^ T cells may have impaired the ability to inhibit tumorigenesis.

A trend toward decreased CD4^+^ T‐cells counts was noted for patients with advancing stage, lymph node metastasis, and distant metastasis, which may imply advancing cancer had escaped immune surveillance and decreased antitumor response.

Naïve CD4^+^ T cells are considered immature and inactivated cells that have not encountered their cognate antigens.^36^ They are activated following encounters with antigen‐presenting MHC class II and become differentiated into effector T cells and long‐lived memory T cells.^37^ It was confirmed that the levels and functions of naïve CD4^+^ T cells were significantly diminished in cancer and HIV patients compared with healthy individuals. The causes of this change are likely multifactorial and secondary to reduced thymic function, increased naïve T‐cell proliferation, enhanced immune activation, and differentiation into other cell subsets.^38^, ^39^ Consistently, we observed lower naïve CD4^+^/CD4^+^ T‐cell percentages in cancer patients than in controls, suggesting that the immune system was activated against cancer and naïve CD4^+^ T cells had differentiated into other cell types.

Memory CD4^+^/ CD4^+^ T cells induce faster and stronger immune responses during secondary encounters with pathogens.^40^ Toward the end of the immune response, the majority of antigen‐specific T cells die and a small percentage differentiate into memory cell subsets.^41^ Increased memory CD4^+^/CD4^+^ T‐cell percentage is a hallmark of adaptive immune memory.^42^ Our results showed that lung cancer patients had higher numbers of memory CD4^+^/CD4^+^ T cells than healthy controls. This result is consistent with other reports.^43^, ^44^ One explanation for this finding is that CD4^+^ T cells were activated and differentiated into memory CD4^+^/CD4^+^ T cells in lung cancer patients.

In summary, significant differences in the levels of NK cells, CD8^+^ HLA‐DR+/CD8^+^ T‐cell percentages, and CD8^+^ CD38^+^/CD8^+^ T‐cell percentages were predictive in all untreated cancer patients and may represent biomarkers for noninvasive early screening in carcinogenesis. CD4^+^ T cell counts and percentages of CD8^+^ HLA‐DR/CD8^+^ and CD8^+^ CD38^+^/CD8^+^ can be potential blood biomarkers of cancer progression.

## CONFLICTS OF INTEREST

The authors declare that they have no conflicts of interest.

## AUTHOR CONTRIBUTIONS

The experiments were conceived and designed by Ying‐Yi Wang, Tai‐Sheng Li, and Chun‐Mei Bai. Sample processing and experiments were performed by Ning‐Ning Li, Chang‐Ting Meng, and Xu‐Hong Zong. Data were analyzed and interpreted by Xiao‐Lei Gong, Zhao Sun, and Xiao‐Yuan Li. The manuscript was written by Ying‐Yi Wang, Na Zhou, Hong‐sheng Liu, and Rui Zhu. All authors reviewed and provided feedback on the manuscript.

## ETHICAL APPROVAL

This article does not contain any studies with animals performed by any of the authors. All procedures were in accordance with the Helsinki Declaration of 1975, as revised in 2013, and followed Macao basic law to protect the privacy of research participants. This study has been approved by ethics committee of CHCSJ (03/CHCSI‐HMEC‐C‐0013‐20114).

## INFORMED CONSENT

Informed consent was obtained from all individual participants.

## Supporting information

Fig S1

Fig S2

## Data Availability

The datasets used and/or analyzed during the current study available from the corresponding author on reasonable request.
