# Supplementary figures and images for: Circulating activated lymphocyte subsets as potential blood biomarkers of cancer progression
Source: Cancer Med. 2020 May 27;9(14):5086–94. doi: 10.1002/cam4.3150 (PMC7367640; doi:10.1002/cam4.3150)

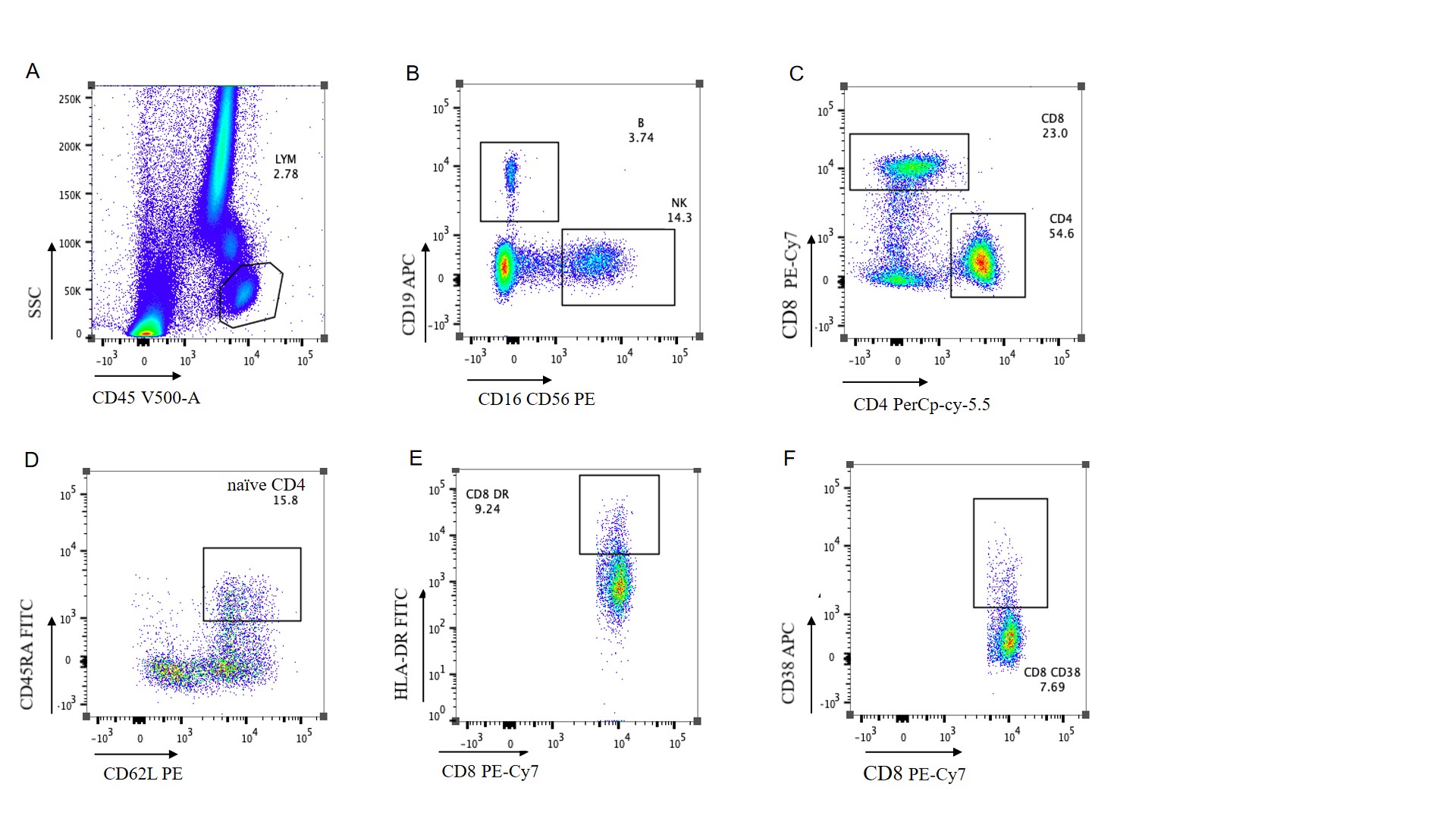

Supplement: Supplementary file 1 — Fig S1 [file CAM4-9-5086-s001.jpg]

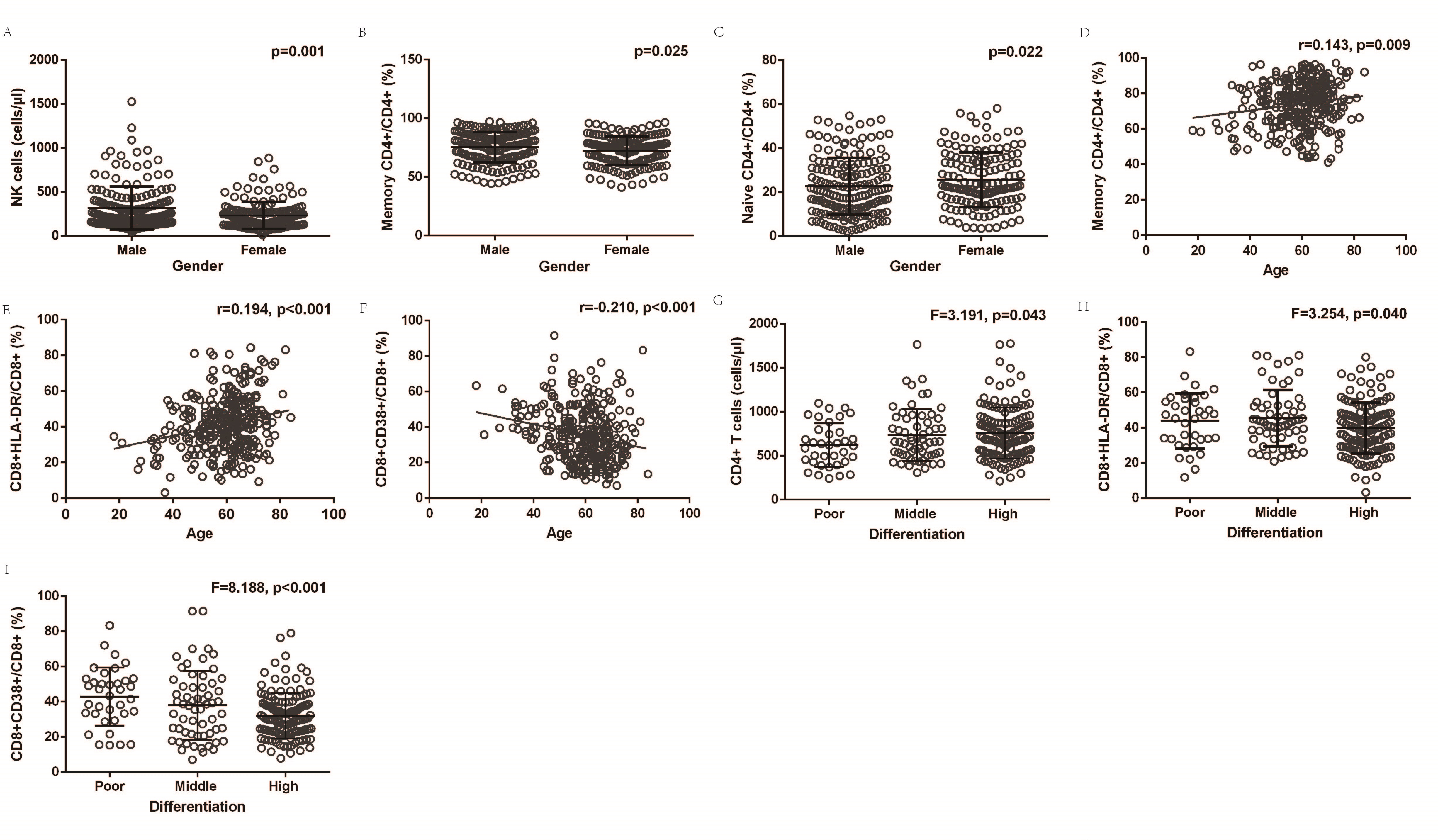

Supplement: Supplementary file 2 — Fig S2 [file CAM4-9-5086-s002.jpg]
